# Dietary fat and carbohydrate modulate the effect of the ATP-binding cassette A1 (*ABCA1*) R230C variant on metabolic risk parameters in premenopausal women from the Genetics of Atherosclerotic Disease (GEA) Study

**DOI:** 10.1186/s12986-015-0040-3

**Published:** 2015-11-16

**Authors:** Leonor Jacobo-Albavera, Carlos Posadas-Romero, Gilberto Vargas-Alarcón, Sandra Romero-Hidalgo, Rosalinda Posadas-Sánchez, María del Carmen González-Salazar, Alessandra Carnevale, Samuel Canizales-Quinteros, Aida Medina-Urrutia, Erika Antúnez-Argüelles, Teresa Villarreal-Molina

**Affiliations:** Laboratorio de Genómica de Enfermedades Cardiovasculares, Instituto Nacional de Medicina Genómica, Mexico City, Mexico; Departamento de Endocrinología, Instituto Nacional de Cardiología “Ignacio Chávez”, Mexico City, Mexico; Departamento de Biología Molecular, Instituto Nacional de Cardiología “Ignacio Chávez”, Mexico City, Mexico; Departamento de Genómica Computacional, Instituto Nacional de Medicina Genómica, Mexico City, Mexico; Laboratorio de Enfermedades Mendelianas, Instituto Nacional de Medicina Genómica, Mexico City, Mexico; Unidad de Genómica de Poblaciones Aplicada a la Salud, Facultad de Química UNAM-INMEGEN, Mexico City, Mexico; Laboratorio de Genómica de Enfermedades Cardiovasculares, Instituto Nacional de Medicina Genómica, Periférico Sur 4809 Colonia Arenal Tepepan, CP 14610 México, D.F. Mexico

**Keywords:** *ABCA1*, R230C, Gene-diet interaction, Insulin resistance, Adiponectin, Visceral to subcutaneous abdominal fat ratio, GGT, HDL-C

## Abstract

**Background:**

Although the R230C-ATP-binding cassette A1 (*ABCA1)* variant has been consistently associated with HDL-C levels, its association with diabetes and other metabolic parameters is unclear. Estrogen and dietary factors are known to regulate *ABCA1* expression in different tissues. Thus, we aimed to explore whether gender, menopausal status and macronutrient proportions of diet modulate the effect of this variant on various metabolic parameters.

**Methods:**

One thousand five hundred ninety-eight controls from the GEA study were included (787 men, 363 premenopausal women and 448 menopausal women), previously assessed for anthropometric and biochemical measurements and visceral to subcutaneous abdominal fat (VAT/SAT) ratio on computed tomography. Taqman assays were performed for genotyping. Diet macronutrient proportions were assessed using a food frequency questionnaire validated for the Mexican population. Multivariate regression models were constructed to assess the interaction between the proportion of dietary macronutrients and the R230C polymorphism on metabolic parameters.

**Results:**

All significant interactions were observed in premenopausal women. Those carrying the risk allele and consuming higher carbohydrate/lower fat diets showed an unfavorable metabolic pattern [lower HDL-C and adiponectin levels, higher VAT/SAT ratio, homeostasis model assessment for insulin resistance (HOMA-IR) and higher gamma-glutamyl transpeptidase (GGT) and alkaline phosphatase (ALP) levels]. Conversely, premenopausal women carrying the risk allele and consuming lower carbohydrate/higher fat diets showed a more favorable metabolic pattern (higher HDL-C and adiponectin levels, and lower VAT/SAT ratio, HOMA-IR, GGT and ALP levels).

**Conclusion:**

This is the first study reporting a gender-specific interaction between *ABCA1*/R230C variant and dietary carbohydrate and fat percentages affecting VAT/SAT ratio, GGT, ALP, adiponectin levels and HOMA index. Our study confirmed the previously reported gender-specific *ABCA1*-diet interaction affecting HDL-C levels observed in an independent study. Our results show how gene-environment interactions may help further understand how certain gene variants confer metabolic risk, and may provide information useful to design diet intervention studies.

**Electronic supplementary material:**

The online version of this article (doi:10.1186/s12986-015-0040-3) contains supplementary material, which is available to authorized users.

## Background

The ATP-binding cassette A-1 protein (ABCA1) is essential for the transport of lipids across plasma membranes and cholesterol homeostasis, is known to have various functions in distinct tissues and plays an essential role in metabolism [1]. ABCA1 is crucial for high-density lipoprotein cholesterol (HDL-C) biogenesis in the liver and intestine [2–4] and is known to regulate insulin secretion in pancreatic β–cells through the modulation of insulin granule exocytosis [5, 6]. Moreover, hepatic ABCA1 was found to improve glucose tolerance by enhancing β-cell function through both HDL production and interaction with β–cell ABCA1 [7], while adipocyte-specific ABCA1 was found to prevent fat storage, and to play a role in adipocyte lipid metabolism, body weight and whole-body glucose homeostasis [8, 9].

The *ABCA1* gene is located at 9q31.1, spans 149 kb, comprises 50 exons and is highly polymorphic. Observations from genetic association studies are in agreement with the role of ABCA1 in HDL-C formation, as several single nucleotide variants (SNVs) have been associated with HDL-C plasma levels in many studies, although their overall contribution to HDL-C levels is low [10–15]. In comparison, few studies have reported associations of *ABCA1* gene variants with other metabolic traits such as type 2 diabetes (T2D) [16–20], body mass index (BMI) [15], or body fat distribution in women [21, 22]. Particularly in the case of T2D, associations with *ABCA1* variants have been inconsistent [23, 24]. Among the many possible explanations for these inconsistencies are the possibility of spurious associations due to population stratification, differences in study design, gender bias and dietary factors known to affect *ABCA1* expression.

Gender-related metabolic differences are well known. *ABCA1* is one of the 1249 sex-biased genes found in liver, expressed at significantly higher levels in females [25]. Some of these gender-related metabolic differences, such as higher HDL-C levels in premenopausal women, can be explained by the effect of estrogen [26, 27]. Both in the murine model and in humans, estrogen has been found to increase *ABCA1* expression in different tissues [28, 29]. Moreover, certain dietary components have been found to regulate *ABCA1* expression: different *ABCA1* transcripts were upregulated by a high fat diet in mice [30]; high glucose, linoleic acid and omega-6 unsaturated fatty acid suppressed *ABCA1* expression while saturated fatty palmitic acid increased *ABCA1* expression in primary human monocyte-derived macrophages [31]; unsaturated fatty acids decreased ABCA1 protein levels by promoting its degradation in HepG2 and human small intestine epithelial cells [32, 33]; ABCA1 hepatic levels were increased in mice fed a high fat diet, while mulberry leaf and fruit extract treatment counteracted this high fat-induced ABCA1 expression [34]; and black soybean supplementation significantly increased hepatic *ABCA1* mRNA levels in mice with diet-induced non-alcoholic fatty liver disease (NAFLD) [35]. Although evidence of dietary factors affecting *ABCA1* expression is abundant, there are relatively few studies reporting of *ABCA1* genetic variants with dietary components. Significant interactions of *ABCA1* gene variants with dietary macronutrients affecting plasma lipid levels have been reported in the Inuit population [36], in healthy young Chinese individuals [37] and in Mexican premenopausal women [38], and two diet intervention studies reported significantly different responses in BMI, HDL-C and serum adiponectin levels in individuals bearing the *ABCA1/*R230C variant [39, 40].

The *ABCA1/*R230C variant (rs9282541) was found to be private to the Americas and strongly associated with low HDL-C in Mexican mestizos and Native American populations [15, 41]. This allele is of particular interest, because it is frequent in the Mexican mestizo population (10 %) and the sole presence of the C risk allele explains almost 4 % of plasma HDL-C concentration variation, which is higher than all HDL-C variation associated with a single nucleotide polymorphism identified through genome-wide association studies in European and Asian populations [42]. Although the *ABCA1*/R230C variant has been consistently associated with HDL-C levels, its association with diabetes and other metabolic parameters is unclear. Thus, we aimed to explore whether gender, menopausal status and dietary macronutrient composition modulate the effect of the *ABCA1/*R230C variant on plasma lipid levels and other cardiometabolic risk parameters.

## Methods

The study included a total of 1598 controls recruited from the GEA Study, which was designed to investigate genetic factors associated with premature coronary artery disease, subclinical atherosclerosis and other coronary risk factors in the Mexican population [21]. All participants provided written informed consent, and the study was approved by the Ethics Committee of the Instituto Nacional de Cardiología “Ignacio Chávez” (INCICH) and the Ethics Committee of the Instituto Nacional de Medicina Genómica (INMEGEN). All participants answered standardized and validated questionnaires to obtain information on family and medical history, menopausal status, alcohol and tobacco consumption, dietary habits and physical activity [43, 44]. Menopausal status was defined as absence of menses for more than 12 months on interrogation. Anthropometric and metabolic measurements included BMI, HDL-C, low density lipoprotein cholesterol (LDL-C), total cholesterol (TC), triglyceride (TG), aspartate transaminase (AST), alanine transaminase (ALT), alkaline phosphatase (ALP), gamma-glutamyl transpeptidase (GGT), adiponectin serum levels, homeostasis model for assessment of insulin resistance (HOMA-IR)], and visceral abdominal fat to subcutaneous abdominal fat (VAT/SAT) ratio on computed tomography scans were measured as previously described [21, 45].

### Dietary assessment

Dietary assessment was performed using a Food Frequency Questionnaire (FFQ) previously validated for the Mexican population by the Instituto Nacional de Salud Pública, using a 116-item semiquantitative FFQ designed to estimate the usual dietary intake over the previous 12-month period [44]. Specifically, this questionnaire was designed to classify individuals by the relative intake of 20 nutrients from 82 foods of the Mexican diet. The FFQ collects specific information on the consumption of fats, carbohydrates, proteins and other nutrients. The validity and reproducibility of this questionnaire to assess dietary intake of a number of nutrients has been assessed previously in this population [44]. Energy intake, and the proportion of macronutrients consumed daily were estimated using the system evaluation of nutritional habits and food intake (SNUT) [46].

### Genetic analysis

Genomic DNA was extracted and purified from white blood cells using the salting-out procedure [47]. The *ABCA1/*R230C variant (rs9282541) was genotyped using TaqMan assay number C_11720861_10, following the manufacturer’s recommendations in an ABI Prism 7900HT sequence detection system (Applied Biosystems). Genotyping call exceeded 95 % and no discordant genotypes were observed in 184 duplicate samples. Previously sequenced samples of all genotypes (RR, RC and CC) were used as positive controls.

### Statistical analyses

Statistical calculations and graphs were made using R [48]. For all calculations, anthropometric and metabolic variables (except age, waist circumference and LDL-C) were log-transformed due to skewed distribution. Genotype frequencies did not deviate from Hardy-Weinberg equilibrium (*P* = 0.57). Because of the reduced number of CC homozygous individuals, RC and CC genotypes were analyzed as a single group using a dominant model. Pearson’s correlations were calculated to study the linear relationship between macronutrient proportions and anthropometric and metabolic variables, stratified by gender, menopausal status and *ABCA1*/R230C genotype. To address multiple testing for the correlation analysis, we calculated Bonferroni’s correction using Simple Interactive Statistical Analyses Software [49] assuming 12 measurements and an average pairwise correlation of 0.21; *P*-value below 0.007 was considered significant. All variables that showed no significant correlation with macronutrients for RR genotypes, but a significant correlation with at least one macronutrient for RC/CC genotypes, were tested for gene-diet interactions. Multivariate regression models were used to assess the interaction between the proportion of dietary components and the R230C variant on the dependent variables and to assess their predictive effect, adjusting for age, BMI and alcohol consumption as appropriate. The study power to detect interactions between the *ABCA1*/R230C variant and dietary fat percentage affecting HDL-C, HOMA-IR, VAT/SAT ratio, adiponectin, ALP and GGT levels in premenopausal women was calculated using QUANTO software version 1.2.4 [50], assuming a dominant model with a minor allele (T) frequency of 0.11 and parameters estimated from the study population. Statistical power ranged between 60 % and 90 % for all variables except for HOMA-IR, which only reached 36 %.

## Results

**Fig. 1:**
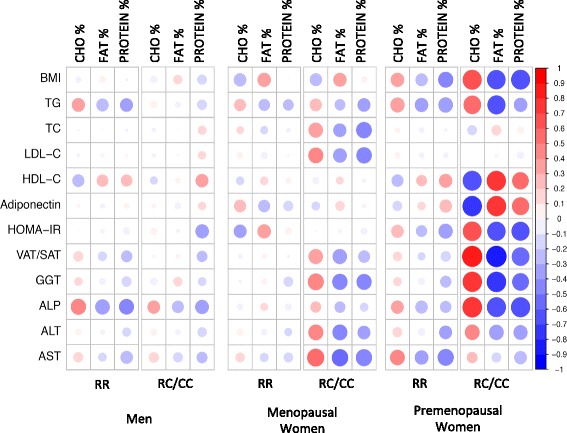
Correlations between macronutrient percentages and metabolic parameters stratified by gender, menopausal status and *ABCA1/*R230C genotypes. Legend: The correlation matrix depicts positive correlations in red and negative correlations in blue. The color scale indicates estimated Pearson’s correlation coefficient values. Statistical significance of each correlation is proportional to the size of the circle

Anthropometric and biochemical characteristics of the population stratified according to gender and menopausal status are shown in Additional file 1: Tables S1 and S2. All correlations between macronutrient percentages and metabolic parameters stratified by gender, menopausal status and *ABCA1/*R230C genotypes are depicted as a correlation matrix in Fig. 1. Overall, correlations between macronutrient percentages and various metabolic parameters were of greater magnitude in women bearing RC and CC genotypes, particularly in premenopausal women. Interestingly, in the latter group, five metabolic parameters (HDL-C, HOMA-IR, VAT/SAT ratio, Adiponectin, and ALP) showed no significant correlation with macronutrients for RR genotypes, but significant correlations with at least one macronutrient for RC/CC genotypes after Bonferroni’s correction (Additional file 1: Table S3). The correlation between dietary fat percentage and GGT levels in RC/CC premenopausal women showed borderline significance (*P* = 0.008) and was also tested for interactions. Median values for these six metabolic parameters according to *ABCA1*/R230C genotypes in the entire study population and in premenopausal women are described in Additional file 1: Table S4. Additional file 1: Tables S5 and S6 show median values for these metabolic parameters according to genotype and stratified by dietary carbohydrate and fat percentage tertiles.

**Fig. 2:**
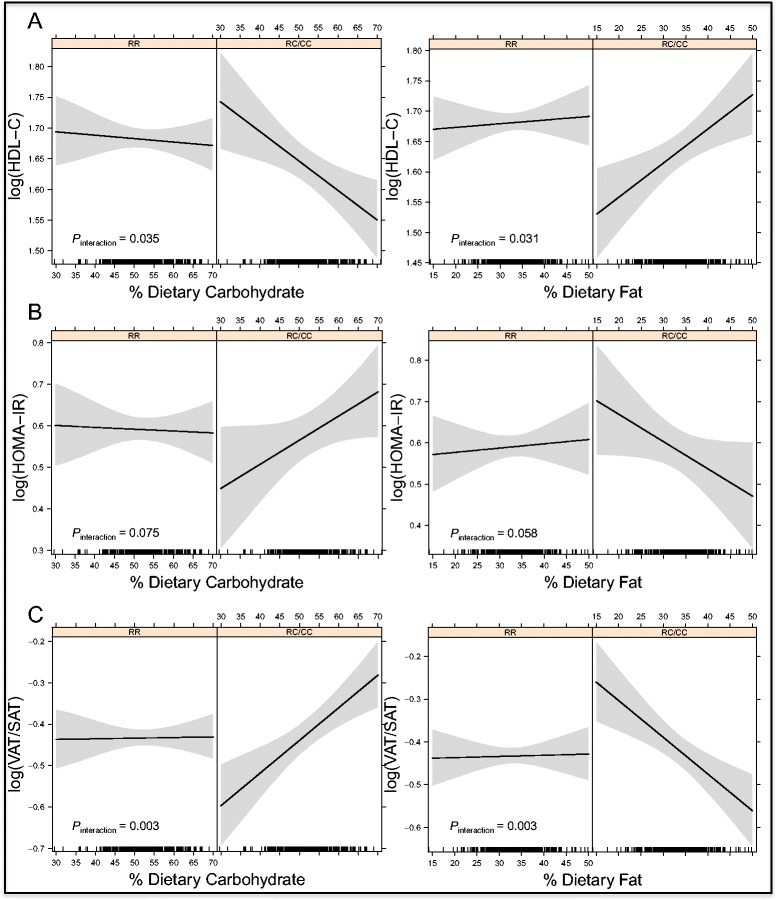
*ABCA1/*R230C-Dietary Macronutrient Interactions Affecting HDL-C Levels, HOMA-IR and VAT/SAT Ratio in Premenopausal Women. Legend: Predicted values for: **a** HDL-C serum levels, **b** HOMA-IR and **c** VAT/SAT ratio in premenopausal women according to the percentage of dietary carbohydrate and fat intake, stratified by *ABCA1/*R230C genotypes under a dominant model (RR = wildtype homozygotes and RC/CC = risk allele heterozygotes and homozygotes)

**Fig. 3:**
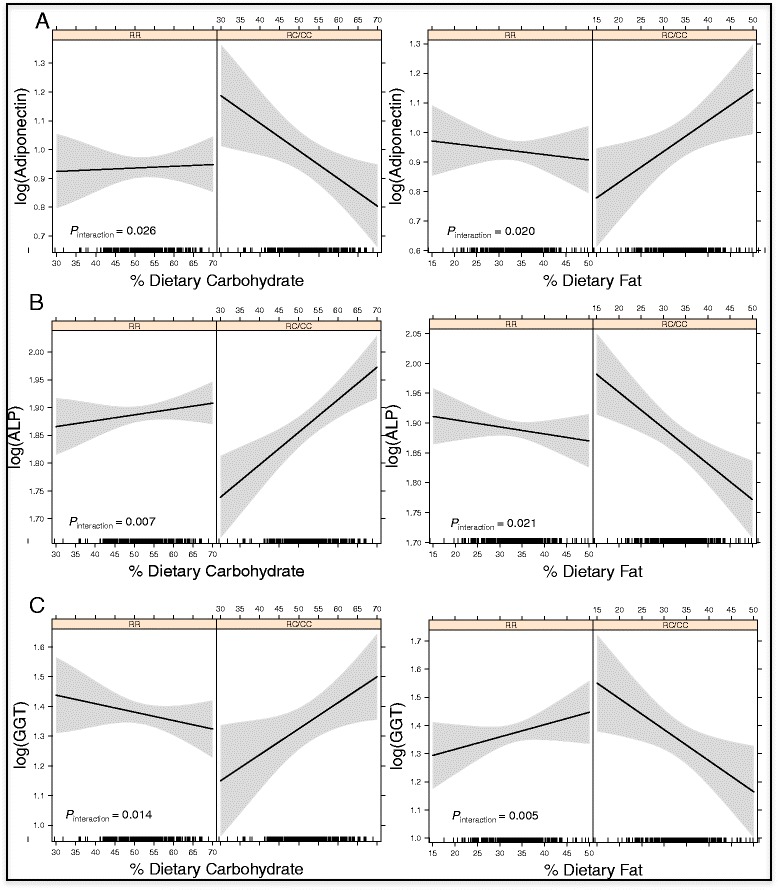
*ABCA1/*R230C-Dietary Macronutrient Interactions Affecting Adiponectin, ALP and GGT Levels in Premenopausal Women. Legend: Predicted values for: **a** serum adiponectin, **b** ALP and **c** GGT levels in premenopausal women according to the percentage of dietary carbohydrate and fat intake, stratified by *ABCA1/*R230C genotypes under a dominant model (RR = wildtype homozygotes and RC/CC = risk allele heterozygotes and homozygotes)

HDL-C and adiponectin serum levels showed negative and significant correlations with dietary carbohydrate percentage and positive significant correlations with fat percentage in premenopausal women with RC and CC genotypes, but not in women with wild-type (RR) genotypes (Additional file 1: Table S3). Both carbohydrate and fat *ABCA1/*R230C interactions affecting HDL-C and adiponectin serum levels showed statistical significance (*P*_*interaction*_ <0.04) (Figs. 2a and 3a).

Correlations between dietary macronutrients and HOMA-IR, VAT/SAT ratio, ALP and GGT showed similar patterns in premenopausal women. All these parameters showed positive and significant correlations with dietary carbohydrate percentage and negative significant correlations with fat percentage in premenopausal women with RC/CC genotypes, but not in women with wild-type (RR) genotypes. Interactions between *ABCA1/*R230C and dietary carbohydrate and fat affecting VAT/SAT ratio, ALP and GGT levels were statistically significant (*P* < 0.03) (Figs. 2c, 3b and 3c); these interactions showed a tendency to significance for HOMA-IR (*P* = 0.075 and *P* = 0.058, respectively) (Fig. 2b). Interestingly, TG levels showed a positive and significant correlation with dietary carbohydrate percentage (*P* < 0.002), and a negative significant correlation with dietary fat percentage (*P* < 0.001) in premenopausal women with RC/CC genotypes, however gene-diet interactions lacked statistical significance.

Dietary protein percentage correlated negatively with ALP levels in premenopausal women with RC/CC genotypes, with borderline significance (*P* = 0.008). This interaction was statistically significant (*P*_*interaction*_ = 0.016).

## Discussion

We present here evidence suggesting that dietary macronutrient proportions modulate the effect of the functional *ABCA1/*R230C gene variant on several metabolic parameters in premenopausal women. Although the total number of premenopausal women was small (n = 363), several gene-diet interactions became evident and significant only in this group. All interactions indicate a metabolically unfavorable pattern in premenopausal women carrying the risk allele consuming higher carbohydrate and lower fat diets (lower HDL-C and adiponectin levels, higher VAT/SAT ratio, HOMA-IR and higher GGT and ALP levels); and a more favorable metabolic pattern in premenopausal women carrying the risk allele with higher fat and lower carbohydrate diets (higher HDL-C and adiponectin levels, and lower VAT/SAT ratio, HOMA-IR, GGT and ALP levels). Gender differences in carbohydrate and fat metabolism are well known [51, 52] and gender differences in metabolic changes in response to diet have been previously published [53–57], mostly evident in women of reproductive age. Although the molecular mechanisms for these differences are not fully understood, evidence both in humans and animal models has shown that the effects of estrogen on genes involved in lipid metabolism play an important role [25–29]. In the GEA control group, *ABCA1*/R230C was significantly associated with HDL-C levels [21], but not with any other metabolic variable. Apparently, the presence of other factors (estrogen levels, low dietary fat, and high dietary carbohydrate) is required for this variant to exert an effect on the other metabolic parameters.

### ABCA1 and serum lipid levels

Many association studies support the role of *ABCA1* gene variation in plasma lipid levels. The vast majority of these studies analyze the role of *ABCA1* polymorphisms on plasma HDL-C levels [10–15]. ABCA1-mediated cholesterol efflux being the first step in HDL-C particle formation likely explains the consistency of the associations of *ABCA1* gene variation with HDL-C levels among different populations. The well-known inverse correlation between dietary carbohydrate intake and HDL-C levels [58, 59] was also observed in control individuals from the GEA study regardless of gender or genotype. However, in agreement with the report of Romero-Hidalgo et al. in an independent Mexican population [38], this inverse correlation was significantly higher in premenopausal women bearing the *ABCA1/*R230C allele (*P*_*interaction*_ *=* 0.04). Also in agreement with this observation, hyperlipidemic R230C allele carriers showed a better HDL-C serum concentration response when fed a low-saturated fat diet supplemented with 25 g soy protein and 15 g soluble fiber, and this response was more evident in women [39], although the total proportion of macronutrients of this diet was not indicated. Thus, three independent studies, two based on food frequency questionnaires and one diet intervention study, confirm a gender-specific *ABCA1/*R230C-diet interaction affecting HDL-C serum levels.

*ABCA1* gene variants have failed to show significant associations with triglyceride levels in almost all genetic association studies analyzing serum lipid levels. To our knowledge, the R230C variant was significantly associated with lower TG and total cholesterol levels in Mayans and Pimas from the United States in only one study [41], and the authors suggested that other genetic or environmental factors may play a role on the effect of ABCA1 on these and other metabolic traits. Interestingly, in the present study although the positive correlation between dietary carbohydrate intake and TG in premenopausal women was higher for RC/CC genotypes (r = 0.366; *P* = 0.002) than for RR genotypes (r = 0.132; *P* = 0.031), and the inverse correlation between dietary fat percentage and TG levels was greater in premenopausal women with RC/CC genotypes (r = -0.384; *P* = 0.001) than in those with RR genotypes (r = -0.122; *P* = 0.046), these interactions lacked statistical significance. A larger study using food frequency questionnaires reported that high saturated fat intake was associated with higher triacylglycerol serum levels in Inuit individuals bearing the CC genotype of *ABCA1/*R219K (rs2230806), with no gender effect reported [36]. Because *ABCA1/*230C allele is less frequent than the *ABCA1/*219K allele, a greater sample size may be necessary to uncover a possible R230C-macronutrient interaction affecting TG levels.

### ABCA1, insulin resistance, VAT/SAT ratio and adiponectin levels

Although several lines of evidence both in human studies and animal models indicate that ABCA1 plays a role in insulin secretion and insulin resistance, the role of *ABCA1* gene variation in T2D susceptibility and insulin resistance is not fully understood. T2D is not a feature of Tangier disease, which is caused by recessive loss of function *ABCA1* mutations. However, while *ABCA1* knockout mice do not develop T2D, pancreatic beta-cell specific *ABCA1* knockout mice *(Abca1*^*-P/-P*^*)* showed a gene-dose dependent and age-related accumulation of cellular cholesterol in β-cells, marked reduction in insulin secretion *in vivo* and progressive impairment in glucose tolerance that was independent of islet development or β-cell mass [5], and disruption of insulin granule exocytosis [6]. Adipocyte specific knockout mice (*Abca1*^-ad/-ad^) also showed impaired glucose tolerance, lower insulin sensitivity and decreased insulin secretion [9].

There are few studies analyzing β-cell function and insulin sensitivity in human heterozygotes for loss-of-function *ABCA1* mutations, with inconsistent results. In agreement with the mouse model, a small study showed that 15 individuals with loss-of function *ABCA1* mutations showed decreased insulin secretion, mild hyperglycemia but no difference in insulin response to an oral glucose challenge measured by hyperglycemic clamp [60]. Because none of these *ABCA1* mutation carriers had diabetes, the authors suggested that carriership is a relatively mild islet susceptibility factor for diabetes by itself. In contrast, in a recent study including only young adults, 3 homozygotes and nine heterozygotes for *ABCA1* mutations exhibited enhanced oral glucose tolerance and increased β-cell secretory capacity as compared to control subjects suggesting a gene dose effect, with no differences in insulin sensitivity [61]. To explain discrepancies with previous studies, the authors suggested that age and systemic cholesterol availability might influence the effect of *ABCA1* mutations on insulin secretion. The largest study of loss-of-function mutations included 94 *ABCA1* heterozygotes from the Copenhagen City Heart Study and the Copenhagen General Population Study. The authors reported no association with increased risk of T2D in the general population, suggesting that ABCA1 dysfunction may impact β-cell function, but is not enough to cause diabetes [24].

It is noteworthy that *ABCA1* has never been identified as a diabetes-associated gene in genome-wide association studies GWAS [62–64]. Associations of *ABCA1* with T2D have only been observed in a small number of candidate gene case–control studies, in Japanese, Mexican and Turkish populations [16–20]. The *ABCA1/*R230C variant was associated with early-onset T2D in two independent small cohorts of the Mexican population [20], was only marginally associated with T2D in Pimas (*P* = 0.06) [41], and was not associated T2D in a case–control study of the Colombian population [23]. Possible explanations for these inconsistencies include differences in study design, gender bias, mean age, population stratification, genetic background, the effect of plasma lipids, and gene-environment interactions, which should be pointed out as possible confounding factors for *ABCA1* association studies. In the present study, it was not possible to directly assess R230C-diet interactions affecting T2D susceptibility because of the low number of premenopausal women with T2D (n = 29). However, interactions affecting T2D-associated metabolic risk parameters (HOMA-IR, VAT/SAT ratio and adiponectin levels) showed the same unfavorable pattern in premenopausal women bearing R230C genotypes with high dietary carbohydrate intake (Figs. 2b, 2c and 3a). To our knowledge, no single *ABCA1* polymorphism has been associated with HOMA-IR or other measures of insulin resistance. The complexity of the process of insulin sensitivity suggests that ABCA1 function is probably only one of many factors affecting glucose uptake by peripheral cells. Although the *ABCA1/*R230C diet interactions affecting HOMA-IR observed here had marginal significance (*P*_*interaction*_ = 0.058 and 0.075 for dietary fat and carbohydrate intake respectively), our data suggest that gender and diet are factors that added to ABCA1 function participate in the complex process of insulin resistance, and deserve further study.

Circulating adiponectin levels are highly heritable [65] and are inversely associated with insulin resistance and T2D [66, 67]. Although the regulation of serum adiponectin levels is complex, experimental evidence suggests there is a link between ABCA1 and adiponectin. Adiponectin has been found to upregulate *ABCA1* expression in macrophages [68, 69] and to play a role in ABCA1 regulation in hepatocytes [70], while the adipocytes of *Abca1*^(ad-/ad-)^ mice were found to have decreased adiponectin expression [9]. Moreover, although *ABCA1* has not been found to be associated with this trait in GWAS or candidate gene association studies, a dietary intervention study including nopal, chia seed, soy protein and oat reported that individuals with metabolic syndrome bearing the *ABCA1/*R230C variant responded with a greater decrease in body weight and a sharper increase in serum adiponectin concentrations [40]. Thus, the significant interactions between the *ABCA1/*R230C variant and dietary fat/carbohydrate percentages observed in premenopausal women from the GEA study support this link between ABCA1 and serum adiponectin levels. Similarly, increased VAT/SAT ratio is associated with increased metabolic risk and T2D [71], and although *ABCA1* has not been associated with VAT/SAT ratio, it was recently identified as a novel locus associated with body fat distribution with a stronger effect in women [22], and BMI was found to be positively associated with VAT/SAT ratio in premenopausal women from the GEA study bearing the *ABCA1/*R230C [21] .

### ABCA1 and liver enzymes

*ABCA1* has not been previously associated with non-alcoholic fatty liver disease, AST or ALT levels, and no gene-diet interactions affecting transaminases were observed in the present study. Interestingly, very significant interactions were observed affecting GGT and ALP serum levels, showing the same unfavorable pattern in premenopausal women bearing the R230C variant with higher dietary carbohydrate and lower dietary fat percentages. In clinical practice, serum GGT has been confirmed to be involved in cardiovascular disease mechanisms, and several studies have reported an independent association of higher GGT levels with metabolic syndrome, carotid atherosclerosis and cardiovascular disease [72–75]. On the other hand, increased GGT and ALP levels are also indicators of liver or biliary tract diseases. ABCA1 is expressed in gallbladder and plays a role in cholesterol concentrations in bile [76, 77], and thus higher ALP and GGT levels observed in premenopausal women bearing *ABCA1/*R230C and consuming high carbohydrates might be related to a higher risk of cholestasis or cholelithiasis. Once again, although *ABCA1* has not been reported as a gene associated with cholelithiasis [78], it might be involved in the pathogenesis of the disease in premenopausal women with certain dietary patterns. Future clinical studies are necessary to prove whether the interactions here observed are in fact related to gallbladder disease.

### Study limitations

Because this is a cross-sectional design, we cannot infer causality from the results. The sample size is relatively small to identify gene-diet interactions, and statistical power is low, particularly to identify interactions affecting HOMA-IR. Further studies are needed to confirm these interactions. One of the main limitations is that carbohydrate and fat percentage were estimated from a validated food frequency questionnaire (FFQ). Dietary intake is difficult to measure, and although doubts of the accuracy of FFQs have been raised, they are still widely used as the primary dietary assessment tool in epidemiological studies [79]. The interactions found based on our FFQ that has been previously validated in the Mexican population can be used to design more controlled and accurate dietary intervention studies. Finally, menopausal status was determined by interrogation, and estrogen levels were not measured.

## Conclusion

To our knowledge, this is the first study reporting a gender-specific interaction between *ABCA1/*R230C variant and dietary carbohydrate and fat percentages affecting VAT/SAT ratio, GGT, ALP and adiponectin levels and HOMA index. Our study also replicated previously reported *ABCA1*-diet interaction affecting HDL-C levels in Mexican premenopausal women observed in an independent study. Our results show how gene-environment interactions may help further understand how certain gene variants confer metabolic risk, and may provide information useful to design diet intervention studies.

## Additional file

Additional file 1:
**Table S1.** Demographic characteristics of the population. **Table S2.** Comparison of biochemical parameters stratified by gender and menopausal status. **Table 3.** Correlation between metabolic parameters and dietary macronutrients according to *ABCA1*/R230C genotypes in premenopausal women. **Table S4.** Comparison of biochemical parameters stratified by *ABCA1*/R230C genotypes in the study population and premenopausal women. **Table 5.** Comparison of biochemical parameters stratified by *ABCA1*/R230C genotypes and carbohydrate percentage tertiles in premenopausal women. **Table 6.** Comparison of biochemical parameters stratified by *ABCA1*/R230C genotypes and fat percentage tertiles in premenopausal women. (DOCX 162 kb)
