# Supplementary material for: Dietary fat and carbohydrate modulate the effect of the ATP-binding cassette A1 (ABCA1) R230C variant on metabolic risk parameters in premenopausal women from the Genetics of Atherosclerotic Disease (GEA) Study
Source: Nutr Metab (Lond). 2015 Nov 16;12:45. doi: 10.1186/s12986-015-0040-3 (PMC4647664; doi:10.1186/s12986-015-0040-3)
Supplement: Additional file 1: — Table S1. Demographic characteristics of the population. Table S2. Comparison of biochemical parameters stratified by gender and menopausal status. Table 3. Correlation between metabolic parameters and dietary macronutrients according to ABCA1/R230C genotypes in premenopausal women. Table S4. Comparison of biochemical parameters stratified by ABCA1/R230C genotypes in the study population and premenopausal women. Table 5. Comparison of biochemical parameters stratified by ABCA1/R230C genotypes and carbohydrate percentage tertiles in premenopausal women. Table 6. Comparison of biochemical parameters stratified by ABCA1/R230C genotypes and fat percentage tertiles in premenopausal women. (DOCX 162 kb) [file 12986_2015_40_MOESM1_ESM.docx]

**Supplementary Table 1. Demographic characteristics of the population.**

|  | **Men** | **Premenopausal women** | **Menopausal women** | ***P*^1^** | ***P*^2^** | ***P*^3^** |
| --- | --- | --- | --- | --- | --- | --- |
|  | **(n=787)** | **(n=363)** | **(n=448)** |  |  |  |
| Age (years) | 53.0 (46.0-60.0) | 47.0 (42.0-51.0) | 58.0 (54.0-63.0) | <0.0001 | <0.0001 | <0.0001 |
| Body Mass Index (Kg/m^2^) | 28.0 (25.7-30.7) | 27.4 (24.9-31.3) | 28.6 (25.7-31.4) | NS | 0.034 | 0.021 |
| Obesity (%) | 29.1 | 30.9 | 36.2 | NS | 0.006 | NS |
| Waist circumference (cm) | 97.5 (91.3-104.5) | 89.4 (82.0-98.5) | 92.2 (84.7-99.7) | <0.0001 | <0.0001 | 0.010 |
| Total Abdominal Fat (cm^2^) | 418.0 (331.0-519.3) | 431.0 (355.5-551.5) | 481.0 (393.8-581.3) | 0.018 | <0.0001 | 0.0002 |
| Subcutaneous Abdominal Fat (cm^2^) | 243.0 (183.0-306.0) | 314.0 (248.5-395.5) | 328.5 (265.0-402.3) | <0.0001 | <0.0001 | NS |
| Visceral Abdominal Fat (cm^2^) | 169.5 (130.0-220.0) | 117.0 (85.0-155.0) | 141.0 (111.0-186.0) | <0.0001 | <0.0001 | <0.0001 |
| Visceral/Subcutaneous adipose tissue ratio | 0.71 (0.55-0.91) | 0.37 (0.30-0.45) | 0.42 (0.33-0.54) | <0.0001 | <0.0001 | <0.0001 |
| Hypertension (%) | 12.8 | 4.1 | 11.0 | <0.0001 | NS | 0.0002 |
| Diastolic Blood Pressure (mmHg) | 73.5 (68.5-80.0) | 68.0 (63.0-73.0) | 70.5 (64.5-76.5) | <0.0001 | <0.0001 | 0.001 |
| Systolic Blood Pressure (mmHg) | 118.0 (110.0-128.0) | 106.0 (100.0-115.0) | 115.0 (105.0-128.0) | <0.0001 | 0.0007 | <0.0001 |

Data are expressed as medians (interquantile range) or percentage. Log-transformed values were used for statistical analysis (except age and waist circumference). Hypertension was defined as systolic blood pressure **≥**140 mmHg and/or diastolic blood pressure **≥**90 mmHg or the use of oral antihypertensive therapy. *P*^1^= men vs premenopausal women, *P*^2^= men vs menopausal women and *P*^3^= premenopausal vs menopausal women; *P*^1^, *P*^2^ and *P*^3^ values were calculated by post-hoc Least Significant Difference (LSD) test for continuous variables and Pearson´s Chi Square test for categorical variables.

**Supplementary Table 2. Comparison of biochemical parameters stratified by gender and menopausal status.**

|  | **Men**  **(n=787)** | **Premenopausal women**  **(n=363)** | **Menopausal women**  **(n=448)** | ***P*^1^** | ***P*^2^** | ***P*^3^** |
| --- | --- | --- | --- | --- | --- | --- |
| Total Cholesterol (mg/dL) | 189.0 (164.0-211.0) | 186.2 (164.0-208.0) | 198.2 (178.0-225.0) | NS | <0.0001 | <0.0001 |
| HDL-C (mg/dL) | 39.9 (34.0-47.0) | 48.0 (38.7-56.0) | 50.2 (41.6-59.5) | <0.0001 | <0.0001 | 0.0006 |
| LDL-C (mg/dL) | 118.6 (97.1-138.0) | 112.1 (93.4-130.0) | 119.8 (100.0-140.8) | 0.036 | 0.028 | 0.0002 |
| Triglycerides (mg/dL) | 160.9 (115.0-225.3) | 132.3 (101.0-185.0) | 144.6 (112.1-199.0) | <0.0001 | 0.0004 | 0.014 |
| ApoA1 (mg/dL) | 124.2 (108.2-143.3) | 135.7 (117.6-159.8) | 146.9 (128.0-167.3) | <0.0001 | <0.0001 | <0.0001 |
| ApoB (mg/dL) | 97.0 (79.0-118.0) | 89.0 (74.5-105.0) | 96.0 (76.0-114.0) | <0.0001 | NS | 0.002 |
| Glucose (mg/dL)^&^ | 90.0 (85.0-96.0) | 86.0 (81.0-93.0) | 89.0 (83.0-96.0) | <0.0001 | NS | 0.0002 |
| HOMA-IR^&^ | 3.7 (2.6-5.4) | 3.5 (2.5-5.2) | 3.8 (2.7-5.3) | NS | NS | NS |
| Adiponectin (μg/mL) | 7.3 (3.9-9.3) | 11.2 (6.0-14.2) | 13.0 (7-1-16.4) | <0.0001 | <0.0001 | 0.001 |
| Alanine Transaminase (IU/L) | 27.0 (20.0-37.0) | 22.0 (16.0-29.0) | 21.5 (16.0-29.0) | <0.0001 | <0.0001 | NS |
| Aspartate Transaminase (IU/L) | 26.0 (21.0-32.0) | 23.0 (19.0-28.5) | 25.0 (21.0-30.0) | <0.0001 | NS | 0.0009 |
| Alkaline Phosphatase (IU/L) | 78.0 (65.0-91.0) | 78.0 (65.0-91.0) | 86.9 (73.0-107.0) | NS | <0.0001 | <0.0001 |
| Gamma-glutamyl transpeptidase (IU/L) | 33.0 (23.0-51.0) | 21.0 (15.0-32.0) | 23.0 (16.0-35.8) | <0.0001 | <0.0001 | NS |

Data are expressed as medians (interquantile range). Log-transformed values were used for statistical analysis (except LDL-C). *P^1^*= men vs premenopausal women, *P^2^*= men vs menopausal women and *P^3^*= premenopausal vs menopausal women; *P^1^*, *P^2^* and *P^3^* values were calculated by post-hoc Least Significant Difference (LSD) test. ^&^Individuals with diagnosis of T2D were excluded from the analysis.

**Supplementary Table 3. Correlation between metabolic parameters and dietary macronutrients according to ABCA1/R230C genotypes in premenopausal women.**

|  | | CARBOHYDRATE % | | FAT % | | PROTEIN % | |
| --- | --- | --- | --- | --- | --- | --- | --- |
|  |  | RR | RC+CC | RR | RC+CC | RR | RC+CC |
|  |  | n= 263 | n=68 | n= 263 | n=68 | n= 263 | n=68 |
| BMI (Kg/m^2^) | r | 0.163 | 0.299 | -0.122 | -0.252 | -0.169 | -0.273 |
|  | *P* | 0.008 | 0.013 | 0.048 | 0.039 | **0.006** | 0.025 |
| TG (mg/dL) | r | 0.132 | 0.366 | -0.122 | -0.384 | -0.079 | -0.102 |
|  | *P* | 0.031 | **0.002** | 0.046 | **0.001** | NS | NS |
| HDL-C (mg/dL) | r | -0.054 | -0.362 | 0.035 | 0.357 | 0.069 | 0.171 |
|  | *P* | NS | **0.003** | NS | **0.003** | NS | NS |
| LDL-C (mg/dL) | r | 0.030 | -0.065 | -0.045 | 0.076 | 0.023 | -0.004 |
|  | *P* | NS | NS | NS | NS | NS | NS |
| TC (mg/dL) | r | 0.074 | -0.077 | -0.098 | 0.065 | 0.023 | 0.067 |
|  | *P* | NS | NS | NS | NS | NS | NS |
| HOMA-IR | r | 0.060 | 0.346 | -0.020 | -0.310 | -0.119 | -0.269 |
|  | *P* | NS | **0.006** | NS | 0.013 | NS | 0.033 |
| VAT/SAT | r | 0.025 | 0.487 | -0.018 | -0.478 | -0.028 | -0.234 |
|  | *P* | NS | **2.58 x 10^-5^** | NS | **3.75 x 10^-5^** | NS | 0.054 |
| ADIPONECTIN (μg/mL) | r | -0.021 | -0.361 | -0.017 | 0.335 | 0.100 | 0.236 |
|  | *P* | NS | **0.004** | NS | **0.007** | NS | 0.062 |
| ALP (IU/L) | r | 0.116 | 0.455 | -0.107 | -0.414 | -0.071 | -0.333 |
|  | *P* | 0.063 | **1.76 x 10^-4^** | NS | **0.001** | NS | 0.008 |
| GGT (IU/L) | r | -0.033 | 0.314 | 0.061 | -0.328 | -0.052 | -0.105 |
|  | *P* | NS | 0.011 | NS | 0.008 | NS | NS |
| AST (IU/L) | r | 0.165 | 0.081 | -0.110 | -0.060 | -0.201 | -0.098 |
|  | *P* | 0.008 | NS | 0.078 | NS | **0.001** | NS |
| ALT (IU/L) | r | 0.052 | 0.228 | 0.003 | -0.255 | -0.150 | -0.026 |
|  | *P* | NS | 0.070 | NS | 0.042 | 0.016 | NS |

r and *P* indicate Pearson’s correlation coefficient and its statistical significance. Log-transformed values were used for statistical analysis (except LDL-C). Bold text indicates a significant *P* value after Bonferroni correction.

**Supplementary Table 4. Comparison of biochemical parameters stratified by ABCA1/R230C genotypes in the study population and premenopausal women.**

|  | **Entire Study Population** | | | **Premenopausal women** | | |
| --- | --- | --- | --- | --- | --- | --- |
|  | RR  (n= 1288) | RC + CC  (n= 297) | **P* | RR  (n= 289) | RC + CC  (n= 72) | **P* |
| HDL-C (mg/dL) | 44.3  (37.0-54.2) | 41.8  (34.0-50.2) | 4.9 x 10^-5^ | 48.7  (40.0-56.3) | 43.3  (35.0-53.5) | 0.019 |
| HOMA-IR | 4.0  (2.7-5.9) | 4.0  (2.6-5.8) | NS | 3.8  (2.5-5.7) | 3.5  (2.6-5.3) | NS |
| VAT/SAT ratio | 0.51  (0.37-0.74) | 0.53  (0.38-0.77) | NS | 0.36  (0.29-0.45) | 0.38  (0.31-0.47) | NS |
| Adiponectin (μg/mL) | 7.8  (4.8-12.2) | 8.4  (5.4-13.0) | NS | 9.2  (5.8-14.3) | 9.7  (6.9-14.2) | NS |
| ALP  (IU/L) | 80.0  (67.0-96.0) | 81.0  (67.2-93.0) | NS | 78.0  (66.0-91.0) | 75.7  (56.0-90.5) | NS |
| GGT  (IU/L) | 28.0  (19.0-43.0) | 25.0  (17.0-41.0) | NS | 22.0  (15.0-32.0) | 19.0  (13.0-31.0) | NS |

Data are expressed as medians (interquantile range). Log-transformed values were used for statistical analysis. **P* values were calculated by t-test.

**Supplementary Table 5. Comparison of biochemical parameters stratified by ABCA1/R230C genotypes and carbohydrate percentage tertiles in premenopausal women.**

|  | **DIETARY CARBOHYDRATE %** | | | | | | | |
| --- | --- | --- | --- | --- | --- | --- | --- | --- |
|  | RR | | | | RC + CC | | | |
|  | Tertile 1  (n= 92) | Tertile 2  (n= 107) | Tertile 3  (n= 68) | *P* | Tertile1  (n= 25) | Tertile 2  (n= 21) | Tertile 3  (n= 21) | *P* |
| HDL-C (mg/dL) | 51.0  (41.6-58.5) | 47.4  (39.0-56.1) | 48.9  (39.9-55.8) | NS | 47.6  (35.8-55.4) | 43.3  (35.6-54.7) | 37.0 (29.7-46.9) | 0.038 |
| HOMA-IR | 3.6  (2.2-5.8) | 4.0  (2.8-6.0) | 3.8  (2.7-5.4) | NS | 3.3  (2.6-5.1) | 3.0  (2.3-4.0) | 4.9  (3.2-8.2) | 0.046 |
| VAT/SAT ratio | 0.35  (0.28-0.46) | 0.37  (0.30-0.44) | 0.37  (0.31-0.44) | NS | 0.33  (0.26-0.41) | 0.38  (0.30-0.49) | 0.46  (0.32-0.62) | 0.011 |
| Adiponectin (μg/mL) | 8.9  (5.3-13.6) | 8.7  (5.7-13.7) | 9.9  (5.7-14.4) | NS | 11.0  (8.7-15.6) | 10.8  (6.2-20.3) | 7.3  (5.6-9.2) | NS |
| ALP  (IU/L) | 74.0  (65.0-86.0) | 79.0  (66.0-96.8) | 82.5  (72.5-93.0) | 0.052 | 66.0  (53.0-85.0) | 63.0  (51.8-91.8) | 86.0  (78.5-123.0) | 0.001 |
| GGT  (IU/L) | 21.0  (15.0-35.0) | 21.5  (16.0-36.0) | 23.0  (15.0-29.0) | NS | 15.0  (11.0-24.0) | 19.0  (12.3-25.3) | 24.0  (17.0-51.5) | 0.030 |

Data are expressed as medians (interquantile range). Log-transformed values were used for statistical analysis. **P* values were estimated using ANOVA.

**Supplementary Table 6. Comparison of biochemical parameters stratified by ABCA1/R230C genotypes and fat percentage tertiles in premenopausal women.**

|  | **DIETARY FAT %** | | | | | | | |
| --- | --- | --- | --- | --- | --- | --- | --- | --- |
|  | RR | | | | RC + CC | | | |
|  | Tertile 1  (n= 66) | Tertile 2  (n= 107) | Tertile 3  (n= 68) | *P* | Tertile 1  (n= 22) | Tertile 2  (n= 17) | Tertile 3  (n= 21) | *P* |
| HDL-C (mg/dL) | 49.0  (39.4-56.2) | 47.4  (38.9-56.4) | 49.7  (41.4-57.3) | NS | 37.5  (29.8-49.2) | 41.9  (35.6-50.8) | 47.1  (35.7-55.6) | NS |
| HOMA-IR | 3.8  (2.6 -5.5) | 3.8  (2.8-5.6) | 3.9  (2.3-5.9) | NS | 4.6  (2.9-8.5) | 3.1  (1.9-4.8) | 3.3  (2.6-4.7) | 0.011 |
| VAT/SAT ratio | 0.39  (0.32-0.45) | 0.37  (0.30-0.44) | 0.35  (0.28-0.45) | NS | 0.44  (0.32-0.60) | 0.38  (0.29-0.51) | 0.33  (0.27-0.41) | 0.007 |
| Adiponectin (μg/mL) | 9.2  (6.5-14.1) | 9.6  (5.7-14.4) | 8.0  (5.1-13.4) | NS | 7.3  (3.7-9.0) | 11.0  (6.9-19.9) | 10.5  (8.5-15.7) | 0.030 |
| ALP  (IU/L) | 83.5  (70.3-94.0) | 78.0  (66.3-91.8) | 77.0  (65.0-87.4) | NS | 85.5  (77.4-123.0) | 61.0  (49.5-99.3) | 66.0  (53.8-85.5) | 0.003 |
| GGT  (IU/L) | 21.5  (15.3-28.0) | 22.0  (16.0-31.8) | 22.0  (15.0-38.0) | NS | 25.0  (17.5-51.3) | 18.5  (13.0-29.8) | 15.0  (11.0-23.3) | 0.023 |

Data are expressed as medians (interquantile range). Log-transformed values were used for statistical analysis. **P* values were estimated using ANOVA.
